# Gait Parameters in Healthy Preschool and School Children Assessed Using Wireless Inertial Sensor

**DOI:** 10.3390/s21196423

**Published:** 2021-09-26

**Authors:** Ewa Gieysztor, Mateusz Kowal, Małgorzata Paprocka-Borowicz

**Affiliations:** Department of Physiotherapy, Faculty of Health Sciences, Wroclaw Medical University, 50-367 Wrocław, Poland; mateusz.kowal@umed.wroc.pl (M.K.); malgorzata.paprocka-borowicz@umed.wroc.pl (M.P.-B.)

**Keywords:** gait analysis, children, wireless inertial sensor

## Abstract

Background: The objective gait assessment in children has become more popular. Basis parameters for comparison during the examination are advisable. Objectives: The study aim was to investigate the typical gait parameters of healthy preschool and school children, using a wireless inertial sensor as the reference for atypical gait. The additional aim was to compare the specific gait parameters in the younger and older group of children. Methods: One hundred and sixty-one children’s gait parameters were evaluated by a G-Walk BTS G-SENSOR smart analyzer. The children were walking barefoot, at a self-selected speed, on a five-meter walkway, and they turned around and go back twice. Results: Age significantly influences most of the spatiotemporal parameters. The support phase becomes shorter with age. Accordingly, the swing phase becomes longer with age. The results also show that older children need shorter double support and have longer single support. Moreover, the pelvic tilt symmetry index is higher with increasing age. In each age division, the smallest variation in all gait parameters within the oldest group of examined children was observed. A comparison between the left and right side gait parameters shows the higher difference in boys than in girls. A significant difference was calculated in the pelvic obliquity symmetry index. Girls had significantly more symmetrical obliquity than boys. Conclusions: the research indicates the basic parameters of typical children’s gait, which may be a reference to atypical gait in the case of trauma or disability.

## 1. Introduction

The latest technology enables the researchers and clinicians to evaluate both neuromotor and biomechanical parameters more precisely. The gait cycle may be observed during the observational part of the evaluation, but in the research, as well as in clinical practice, the objectivity of the measurement is valuable, so the evaluation using the special dedicated instrument is very important. Gait is one of the most commonly assessed ways of investigating human movement. The development of gait rises with age, and in children of about 7 years old, it seems to be mature [1,2]. However, the difference in its pattern over the years is observed in the research [3,4,5]. The evaluation of gait is especially important in the abnormal ways of moving during illness or disability; nevertheless, the typical way of moving must be the basic indication for comparison in changed conditions.

There are many articles describing gait being connected with different disabilities, such as cerebral palsy, amputation, or Charcot–Marie–Tooth disease, as well as a neuromotor delay [6,7,8,9,10,11,12,13]. Since typically developed children move in a different way, the normative data for this group of age are also desirable [5,14,15].

Taking the above into consideration, the basic aim of the study was to investigate the normal gait parameters in Polish children. Moreover, the differences in gait parameters in preschool children vs. school children during walking were investigated, as well as in the annual division. The gait parameters of healthy children may be the basis for analyzing the differences in atypical gait parameters.

## 2. Materials and Methods

The research has complied with all relevant national regulations and institutional policies, following the tenets of the Declaration of Helsinki. The study was approved by the Wroclaw Medical University’s Ethical Committee KB-116/2019. All parents of the subjects were kept informed of the purpose and process of examination and had given their written consent prior to the study.

### 2.1. Participants

The participants were recruited from preschools and schools in Wrocław (Poland). Inclusion criteria were typically developing children of both sexes aged 3.5–12 years and with the ability to follow verbal instructions. Exclusion criteria were as follows: any known neuromuscular and/or orthopedic, neurological or developmental disorders, inability to follow directions. Moreover, using assistive devices, or current or past balance disorders were also the parameters excluded from the research. In the examination 161 children took part. There were 79 girls and 82 boys.

### 2.2. Protocol of Gait Analysis

The applied method enabled some parameters to be collected regarding gait biomechanics of normal children, such as spatiotemporal parameters as well as symmetry of motion of pelvis.

Gait analysis was performed using a BTS G-SENSOR measurement instrument (BTS Bioengineering Corp., Quincy, MA, USA). The device was equipped with triaxial accelerometer 16 bit/axes with multiple sensitivities (±2, ±4, ±8, ±16 g), triaxial gyroscope 16 bit/axes with multiple sensitivities (±250, ±500, ±1000, ±2000 °/s), as well as triaxial magnetometer, 13 bit (±1200 uT). An inter-instrument correlation coefficient between 0.90 and 0.99, and an intra-instrument coefficient of variation of ≤2.5% found the G-SENSOR to be suitable for the assessment of physical activity [16,17].

The measurement was conducted in their school to provide natural conditions as this was an environment known to the children. Anthropometric measurements, such as body height (m), body weight (kg) and leg length (cm) (from the greater trochanter to the floor), were collected. Afterwards, each child had a wireless inertial sensor fixed using a semi-elastic belt at the lower lumbar level, centered on the L4–L5 intervertebral disc. Participants were then instructed to walk barefoot in the most natural way for them at a self-selected speed on a five-meter walkway, turn around and go back, twice. The raw data were then processed with the BTS G-SENSOR (BTS Bioengineering, Corp., Quincy, MA, USA) dedicated software to calculate the following spatiotemporal parameters: cadence (steps/min), velocity (m/s), step length (m), swing and double support phase duration (calculated as a percentage of the gait cycle). Moreover, to measure symmetry of pelvis movement during walk, the following were taken into account:

- Pelvic obliquity: upward (positive) or downward (negative) movement of the pelvis in the frontal (F) plane;
- Pelvic tilt: anterior or posterior movement of the pelvis in the sagittal (S) plane;
- Pelvic rotation: internal (positive) or external (negative) movement of the pelvis in the transversal (T) plane.

### 2.3. Statistical Analysis

Statistical analysis was performed using Statistica Version 13.3. Arithmetic means and standard deviations were calculated for continuous variables. In order to determine the relationship between quantitative variables, Spearman’s rho correlation analysis was used. The *t*-test and Mann–Whitney U test were used to compare groups in terms of nominal/categorical variables. The level α = 0.05 was used for all comparisons.

## 3. Results

The results were described as correlation coefficients, as well as means, standard deviations, and medians of spatiotemporal parameters of gait. The investigation was also conducted in divisions, due to age and sex.

### 3.1. Participants’ Demographics

The number of participants, age, weight, height, and BMI characteristics, in the division of sex, are detailed in Table 1.

The mean age of the group was 6.82 (±2.13) years. The mean body height of the children was 1.21 m (±0.14). The mean weight was 24 kg (±7.4) in the group. The mean BMI was 16 kg/m^2^. The children were examined in two groups, divided depending on their age. The preschool (P) children were 3.5–6 years old (*n* = 70), and the school children (S) were over 6 years old (*n* = 91). The groups differed in age, height, and weight. The BMI of the children was normal in the two groups. The characteristics of the children, depending on the age groups, are presented in Table 2.

**Figure 1:**
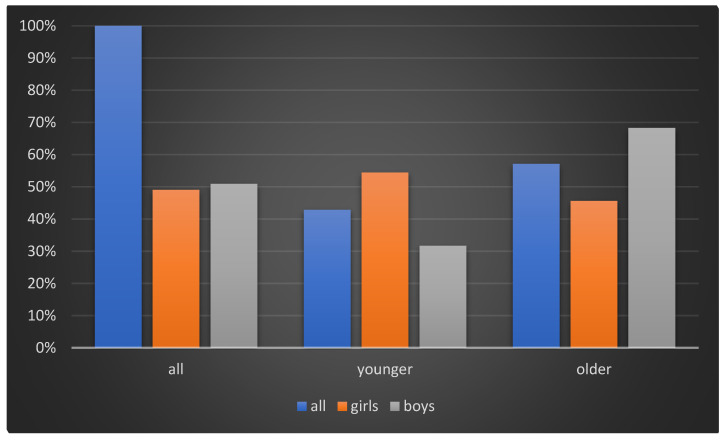
Histogram of the percentage of children in examined group with sex division.

**Figure 2:**
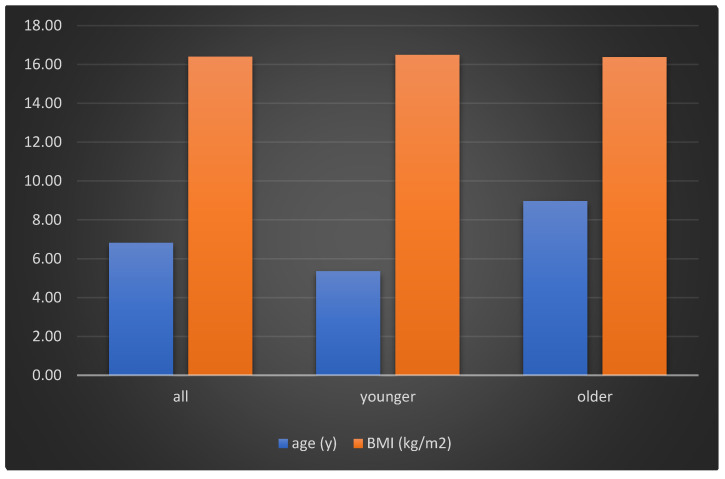
Histogram of the age and BMI distribution in the whole group and in the division of age groups.

The distribution of sex, age, and BMI is shown in Figure 1 and Figure 2. The data show the distribution in the following three ways: the whole examined group, and in the division of younger and older groups of children. The older group had more children and there were more boys in this group compared to the younger group.

### 3.2. Spatiotemporal Gait Parameters

The detailed spatiotemporal parameters are shown in Table 3. All the parameters, excluding standardized step length, are shown.

### 3.3. Correlation Coefficient Investigation

The parameters of gait were analyzed using a correlation coefficient. As a result, age and some gait parameters were found to be related. Cadence was negatively correlated with age, which means that younger children do more steps per minute. Moreover, the support phase duration was negatively correlated with age. This was interpreted from the fact that the support phase becomes significantly shorter with age (−0.31; −0.42). Accordingly, the swing phase becomes longer with age (+0.31; +0.42). The results also show that older children need shorter double support (−0.31) with longer single support (+0.44; +0.32). Moreover, the pelvic tilt symmetry index is higher with age. No significant correlations were calculated for BMI in the examined group of children. The results are shown in Table 4.

### 3.4. Gait Parameters Due to Annual Division

**Figure 3:**
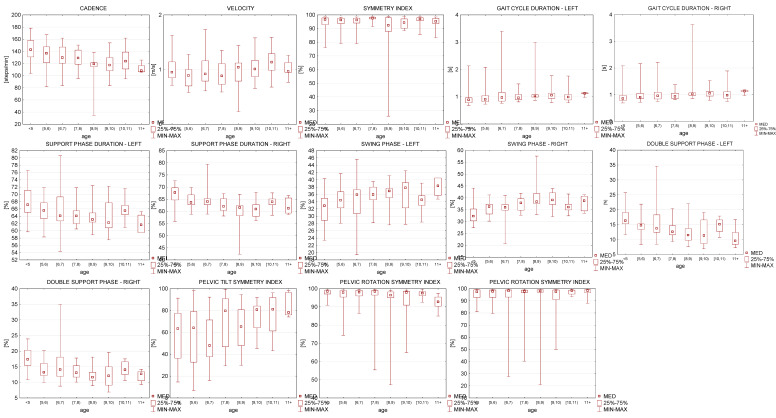
Normative reference gait parameters. Spatiotemporal gait metrics in children. All data reported as median, minimum and maximum.

The parameters were also analyzed in the divisions of each age. Figure 3 shows the median, minimum, and maximum of the results. On the diagrams, the variability in gait parameters, depending on age, is shown. Some of them have the tendency to increase with age, such as swing phase and pelvic tilt symmetry. Whereas, the pelvic rotation and obliquity symmetry index appear to be at a constant level. We observed the smallest variation of all the gait parameters in the oldest group of examined children.

### 3.5. Gait Parameters Due to Preschool and School Groups

The group of examined children was divided into a younger and older group. Accordingly, under and over six years old. For the groups, the gait parameters were compared using a *t*-test, and the correlation coefficient was calculated.

The results are shown in Table 5.

The statistical difference between the groups was observed in the cadence parameter (*p* < 0.001). So, younger children have significantly less-efficient gait. The velocity and step length parameters were higher in the older group, but the differences were not statistically significant.

### 3.6. Differences between Left and Right Side in the Gait Parameters

Moreover, the difference between the left and right side parameters was calculated. A statistical difference was found in the support phase duration (*p* = 0.001) and swing phase (*p* = 0.001) for all the children. In terms of sex division in both the groups, girls and boys, a significant difference between the left and right side was observed in support phase duration (girls *p* = 0.04, boys *p* = 0.01), swing phase (girls *p* = 0.04, boys *p* = 0.01), and single support duration (girls *p* = 0.04, boys *p* = 0.01).

In age division, a significant difference between the sides was observed in the older group, for support phase duration (*p* = 0.001), swing phase (*p* = 0.001), and single support duration (*p* = 0.001). The differences were not observed in the younger group of children.

### 3.7. Gait Parameters Divided into Two Groups Due to Sex

An analysis of the differences between girls and boys was performed using *t*-test. The only significant difference was calculated in the pelvic obliquity symmetry index. Girls had significantly more symmetrical obliquity than boys (*p* = 0.03).

## 4. Discussion

The objective of this study was to compare multiple gait parameters between two typically developed age groups of children, as well as year-by-year age parameters. The age of the groups was 6 years old. This is the point at which the younger children are in the group of preschool and the older are in school. It seems to be the natural division, especially taking into consideration the expectation of gait maturity.

In the study, a correlation of the gait parameters was only found with age. BMI and sex do not significantly impact gait. The older children needed shorter double support, and single support in this group was longer, as well as swing phase; whereas Voss et al. [18], in their study, present that age affected all the gait parameters, except double support time. The results in our research may be explained by the increased level of balance with age [19]. An equilibrium recovering ability takes a specific role in the maturation of the gait pattern. The symmetry in pelvic tilt with age was the next parameter that increased with age.

Manicolo et al. [20] show the gait variability in children with neuro-maturity disorders. Their work describes less regular gait patterns in the group of ADHD children. Moreover, the authors emphasized that the results indicate a maturation delay rather than a permanent gait deviation. They also show that the gait patterns become more regular with age, as was shown in our work.

Cadence was the next parameter that improved with age. The correlation coefficient, as well as *t*-test comparison, showed the significant difference between preschool children and school children. In the research by Papadopoulos et al. [21], on ADHD children, the results of gait analysis during a self-selected fast speed show that they had higher cadence and walked faster than the control group. The study indicates some alteration in gait patterns. As the work shows gait parameters in atypically developed children, the comparison cannot be strict; however, it indicates that disturbances in development impact cadence. It may also show that ADHD children’s gait parameters behave similarly to typical younger children. Voss et al. [18] had similar results to our findings, as they found that cadence decreased progressively with age.

In our study, we firstly divided children into two groups depending on age. The results show that walking differs significantly in the groups of preschool children and school-age children. Most of the gait characteristics were statistically different, which, in a clinic, suggest that younger children’s gait parameters should not be compared to older children’s, as well as indicating that intense carefulness during standard clinical examination is needed. Analyzing the correlation coefficient between gait and age, the results show that most of the gait parameters were affected by age.

The similarity of gait was investigated using a wide range of instruments and computer technology for the observation and measurement of the human walk. They are deeply analyzed in the paper of Viteckova et al. [22]. In our work, the comparison of the difference between the right and left lower limbs gait parameters showed that the support phase duration, swing phase, and single support duration differ in terms of sex and in the older group. There were no significant differences between sides in the younger group. This means that side differentiation (lateralization), which is typically observed as complete at school age, may also be observed in gait patterns. Since the asymmetry in gait is usually analyzed in the case of orthopaedical or neurological injury, this kind of asymmetry shows biomechanical disturbance instead of normal gait [23,24]. This topic, for typically developed children, should be investigated more carefully in future research. As we did not find research showing similar findings, a comparison is not possible.

Analyzing sex impact on gait, we found no correlation. The only significant difference was visible in the pelvic obliquity symmetry index. Girls had significantly more symmetrical obliquity than boys. Similar results were shown by Moreno-Hernandez et al. [25], who found no significant differences between sexes. They claim that the differentiation in gait patterns may depend on footwear. The use of footwear caused increased velocity, cadence, step, and stride length, while the percentage of stance phase was reduced. This can be an idea for future investigations, to check the gait in different footwear as well. Additionally, Voss et al. [18] found that sex only impacted cadence and normalized gait speed.

Another work, which analyzed gait in children, was by Lythgo et al. [26]. They described a similar group of participants (5 to 13 years old) and assessed the gait at three different speeds. They indicated a significant speed differentiation between slow, free, and fast walking. The examined pupils walked 24% slower and 30% faster than the free speed condition. In the context of the results of the other researchers, it encourages us to conduct the gait study in the group of children in various conditions, such as speed, environment, and footwear. The comparison may bring a wider image of the gait in a typically developed group of children.

All the studies used for this discussion can only point to similarities in the gait, but in spite of the different points of view, the comparison cannot be strict [27]. All the aforementioned measurements were conducted using different programs for movement analysis. The available literature does not allow the strict comparison of the results, because of the different ways of collecting data; however, it does paint a particular picture of this topic, which is important for research and clinical reasons.

### Limitations of the Study

The gait analysis was only based on measurements of time-spatial variables of gait and does not provide a complete picture of movement analysis in children. Future research could include measuring changes in the range of motion between the pelvis, torso, and skull/head segments. An advantage of the presented examination, however, may be that it is clinically viable and tangible.

## 5. Conclusions

There are differences in the gait parameters in younger and older groups of children, which should be considered in the clinical evaluation. The results show that age significantly influences most of the spatiotemporal parameters. The support phase becomes shorter with age. Accordingly, the swing phase becomes longer with age. The results also show that older children need shorter double support and have longer single support. Moreover, the pelvic tilt symmetry index becomes higher with age. In each age division, the smallest variation within all the gait parameters was observed in the oldest group of examined children.

The results of the study may be useful in diagnostic and rehabilitation programs as the basal parameters.

The normative parameters can be important in comparison with the group of atypical gait patterns in the case of disability.

## Figures and Tables

**Table 1:** Characteristics of the group. Whole group characteristics as well as in division of sex. The data are described as means and standard deviations.

	All	P Group	S Group
***n***			
Female	79 (49%)	43 (61%)	36 (40%)
Male	82 (51%)	27 (39%)	55 (60%)
Total	161	70	91
**Age (y)**			
Female	7.16 (±2.15)	4.93 (±0.76)	8.29 (±1.56)
Male	6.46 (±2.06)	4.89 (±0.75)	8.24 (±1.71)
Total	6.82 (±2.13)	4.91 (±0.75)	8.26 (±1.64)
**Weight (kg)**			
Female	22.65 (±6.44)	19.19 (±4.30)	26.78 (±6.15)
Male	25.98 (±7.94)	20.26 (±3.71)	28.68 (±7.99)
Total	24.34 (±7.40)	19.60 (±4.09)	27.93 (±7.34)
**Height (cm)**			
Female	118.56 (±15.08)	108.07 (±7.65)	131.08 (±11.83)
Male	123.00 (±13.40)	110.19 (±5.89)	129.05 (±11.55)
Total	120.81 (±14.38)	108.87 (±7.07)	129.86 (±11.64)
**BMI (kg/m^2^)**			
Female	15.96 (±2.64)	16.38 (±2.97)	15.45 (±2.11)
Male	16.88 (±2.75)	16.69 (±2.78)	16.98 (±2.72)
Total	16.43 (±2.72)	16.50 (±2.88)	16.37 (±2.60)

**Table 2:** Characteristics of the groups. Differences between younger (P group) and older (S) group.

	P Group	S Group	*p*-Value
mean age (y)	5.35 (±0.97)	8.95 (±1.4)	*p* < 0.001
mean body height (m)	1.12 (±0.8)	1.34 (±0.11)	*p* < 0.001
mean body weight (kg)	21 (±4)	30 (±8)	*p* < 0.001
BMI (kg/m^2^)	16.5	16.4	*p* > 0.05

**Table 3:** Spatiotemporal gait parameters in examined group. *p*-value is described for difference between P and S in means.

		All Children			Preschool			School			
Spatiotemporal Gait Parameters											
		Mean	SD	Median	Mean	SD	Median	Mean	SD	Median	*p*-Value
gait cycle duration (s)	left	0.93	0.20	0.89	0.85	0.17	0.80	0.99	0.20	0.96	0.000
	right	0.92	0.19	0.89	0.84	0.17	0.80	0.98	0.19	0.97	0.000
step length (%)	left	75.79	17.43	73.10	75.81	21.51	72.70	75.77	13.51	73.70	0.987
	right	75.50	17.18	73.90	75.63	21.65	73.30	75.39	12.76	74.10	0.931
support phase duration (%)	left	65.54	4.27	65.10	67.13	4.26	66.30	64.33	3.86	63.50	0.000
	right	64.03	3.91	63.55	65.94	4.06	66.10	62.59	3.09	62.50	0.000
support phase duration (%)	left	65.54	4.27	65.10	67.13	4.26	66.30	64.33	3.86	63.50	0.000
	right	64.03	3.91	63.55	65.94	4.06	66.10	62.59	3.09	62.50	0.000
swing phase (%)	left	34.46	4.27	34.90	32.87	4.26	33.70	35.67	3.86	36.50	0.000
	right	35.97	3.91	36.45	34.06	4.06	33.90	37.41	3.09	37.50	0.000
double support duration (%)	left	14.59	4.02	14.35	16.23	3.42	15.50	13.35	4.00	12.70	0.000
	right	14.62	3.83	14.10	16.27	4.17	16.10	13.37	2.99	12.90	0.000
single support duration (%)	left	36.26	3.89	36.70	34.48	3.79	34.30	37.61	3.38	37.70	0.000
	right	34.81	3.96	35.40	33.45	4.02	33.60	35.85	3.58	36.50	0.000
Gait symmetry index (%)											
		Mean	SD	Median	Mean	SD	Median	Mean	SD	Median	*p*-value
pelvic tilt (S)		62.55	24.31	68.80	55.69	25.76	62.70	67.76	21.76	72.00	0.002
pelvic obliquity (F)		96.00	5.83	98.00	96.83	4.24	98.50	95.37	6.72	97.50	0.117
pelvic rotation (T)		94.68	9.94	97.95	94.53	9.51	97.70	94.79	10.24	98.10	0.868

**Table 4:** Correlation between age, BMI and gait parameters.

		Age	BMI
cadence (steps/min)		−0.43*	0.17
velocity (m/s)		0.12	0.09
gait cycle duration (s)	left	0.10	−0.04
	right	0.12	−0.06
step length (%)	left	−0.06	0.04
	right	−0.06	0.05
support phase duration (%)	left	−0.31*	0.15
	right	−0.42*	0.08
swing phase (%)	left	0.31*	−0.15
	right	0.42*	−0.08
double support duration (%)	left	−0.38*	0.10
	right	−0.38*	0.10
single support duration (%)	left	0.44*	0.00
	right	0.32*	−0.13
pelvic tilt (S)		0.32*	−0.03
pelvic obliquity (F)		−0.17	−0.08
pelvic rotation (T)		−0.01	−0.05

*p*<0.05

**Table 5:** Characteristic of cadence, velocity and step length in all examined groups as well as in age groups division.

		Cadence (Steps/min)	Velocity (m/s)	Step Length (cm)
all children	mean	130.99	0.99	94.91
	SD	19.89	0.58	4.37
	min	82.10	0.58	76.10
	max	178.30	1.73	99.00
preschool	mean	139.37	0.96	94.70
	SD	20.55	0.24	4.76
	min	82.10	0.58	76.10
	max	178.30	1.63	98.70
school	mean	124.64	1.01	95.07
	SD	16.78	0.25	4.04
	min	83.60	0.59	79.00
	max	161.90	1.73	99.00

## Data Availability

Not applicable.
